# Supplementary material for: Mammographic density mediates the protective effect of early-life body size on breast cancer risk
Source: Nat Commun. 2024 May 13;15:4021. doi: 10.1038/s41467-024-48105-7 (PMC11091136; doi:10.1038/s41467-024-48105-7)
Supplement: Supplementary file 1 — Supplementary Information [file 41467_2024_48105_MOESM1_ESM.pdf]

Supplementary materials

For the article: **Mammographic density mediates the protective effect of early-life body size on breast cancer risk** (Vabistsevits et al 2024)

In this document:

- Supplementary Tables 1 and 2
- Supplementary Note 1 (MR results using MD GWAS adjusted for BMI)
    - Supplementary Figures 1-3
- Supplementary Figures 4-9
- Supplementary Note 2 (Mediation analysis)
    - 2.1 DA all instruments
    - 2.2 DA cluster_2 instruments (outlier-corrected IVW)
- Supplementary Note 3 (outlier-corrected IVW for breast cancer subtypes)

Supplementary Data 1-19, 20-25, 26-37 are provided in separate xlsx files.

Data 1-12 and 26-37 are equivalent; the former contains results for the unadjusted MD data (as in the main results), while the latter results are based on MD data adjusted for BMI (as in [1]).

Supplementary Table 1. Summary of breast cancer GWAS datasets from the BCAC 2017 [2] and BCAC 2020 [3] releases. Sample sizes for BCAC 2020 were taken from [4].

Release	Subtype	Receptor/grade status	Sample size	Cases	Controls	% cases
2017	Overall sample 2017		228,951	122,977	105,974	53.7%
	ER+	ER+	175,475	69,501	105,974	39.6%
	ER-	ER-	127,442	21,468	105,974	16.9%
2020	Overall sample 2020		247,173	133,384	113,789	54.0%
	Luminal A	ER+ and/or PR+, HER2−, grades 1 and 2;	155,244	63,767	91,477	41.1%
	Luminal B1	ER+ and/or PR+, HER2+;	107,419	15,942	91,477	14.8%
	Luminal B2	ER+ and/or PR+, HER2−, grade 3;	107,419	15,942	91,477	14.8%
	HER2-enriched	ER− and PR−, HER2+	102,105	10,628	91,477	10.4%
	TNBC	ER− and PR−, HER2−	100,079	8,602	91,477	8.6%

Supplementary Table 2. The number of MD phenotypes' genetic instruments in unadjusted GWAS data (primary dataset) and adjusted for BMI data (dataset used for comparison only; corresponds to data published in [1]).

	**Unadjusted data**			**Adjusted for BMI data**		
	Total N after extraction	N in BCAC outcome 2017 / 2020		Total N after extraction	N in BCAC outcome 2017 / 2020	
**Dense area**	21	20	20	25	23	22
**Non-dense area**	8	7	8	16	15	16
**Percent density**	11	11	11	16	15	16

Supplementary Note 1. MR results when using MD GWAS adjusted for BMI

1) Effect of childhood/adult body size on MD

When using the MD GWAS data adjusted for adult BMI (originally published in Sieh *et al* (2020) [1]), we observed an attenuation in the MVMR results (Supplementary Figure 1, Supplementary Data 28) of adult body size on all MD phenotypes, which may have suggested that childhood body size affects MD independently of adult body size.

This result, however, was a consequence of adjustment for BMI in the original GWAS summary statistics, leading to a double adjustment in MVMR with adult body size. This is a good example of how adjusted GWAS data in MR may create misleading results [5][6]. As such, we used the MD GWAS unadjusted for BMI for all primary analyses undertaken in this study.

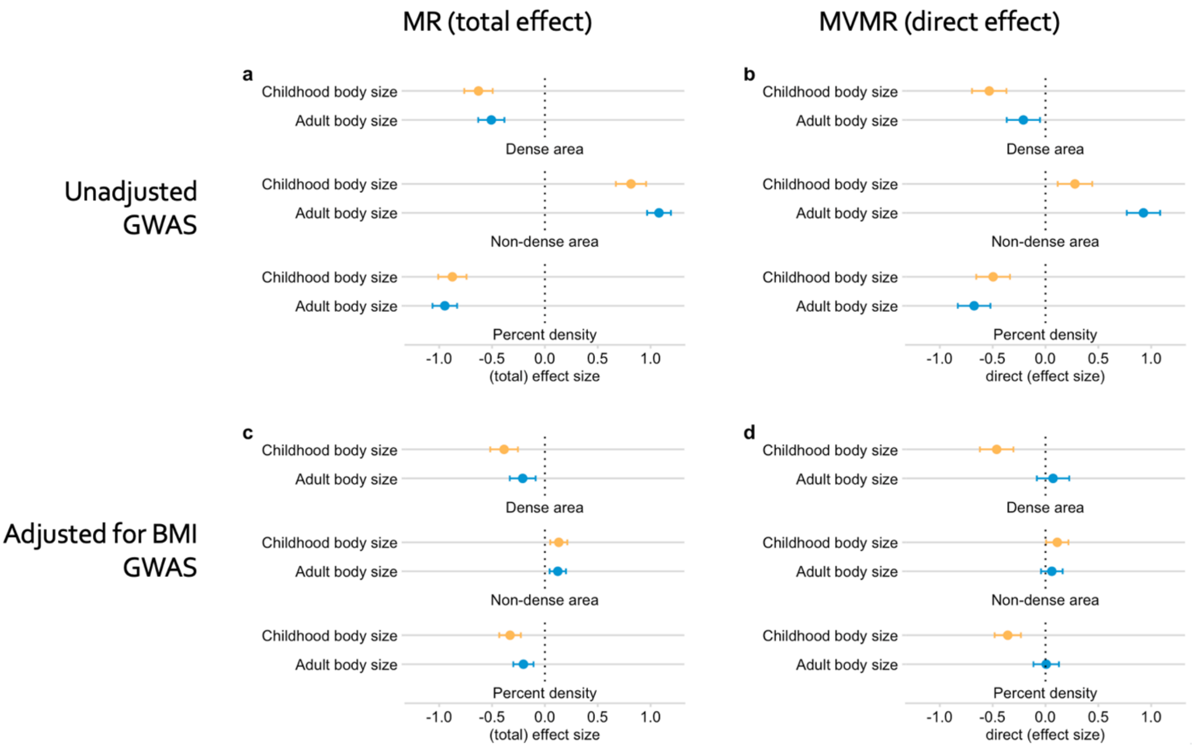

Supplementary Figure 1. The effect of childhood body size and adult body size on MD phenotypes (dense area, non-dense area, percent density), when using **unadjusted** MD GWAS (a, b) and **adjusted for BMI** MD GWAS (c, d). The results are presented for univariable MR total effect of each exposure on the outcome (a, c), and MVMR direct effect (b, d). Bars indicate 95% confidence intervals around the point estimates from IVW analyses.

2) Effect of age at menarche on MD

Age at menarche had no effect on MD (DA and PD) when using the MD GWAS adjusted for BMI (Supplementary Figure 2, Supplementary Data 30).

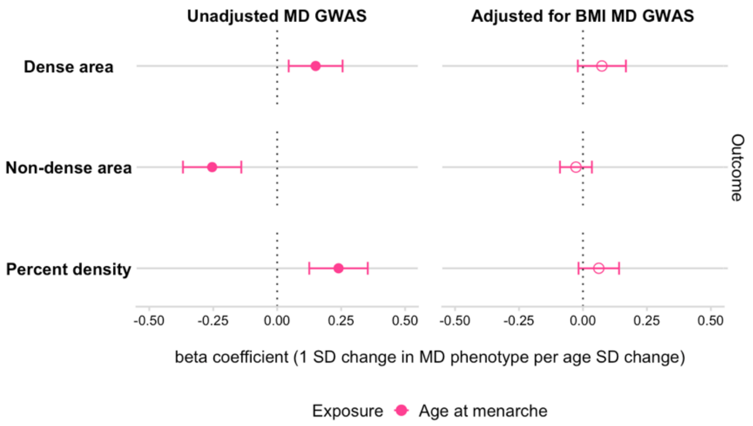

Supplementary Figure 2. The effect of age at menarche on MD phenotypes (dense area, non-dense area, percent density), when using **unadjusted** MD GWAS and **adjusted for BMI** MD GWAS. Bars indicate 95% confidence intervals around the point estimates from IVW analyses. The empty circle data points highlight the results where confidence intervals overlap the null.

3) Effect of MD on breast cancer

The comparison of results using the BMI-adjusted and unadjusted MD GWAS are presented in Supplementary Figure 3 and Supplementary Data 34. When using the adjusted data, a few more instruments were available for the analysis (Supplementary Table 2), however, there was similar heterogeneity among the instruments and imprecise estimates of effect on the majority of breast cancer subtypes.

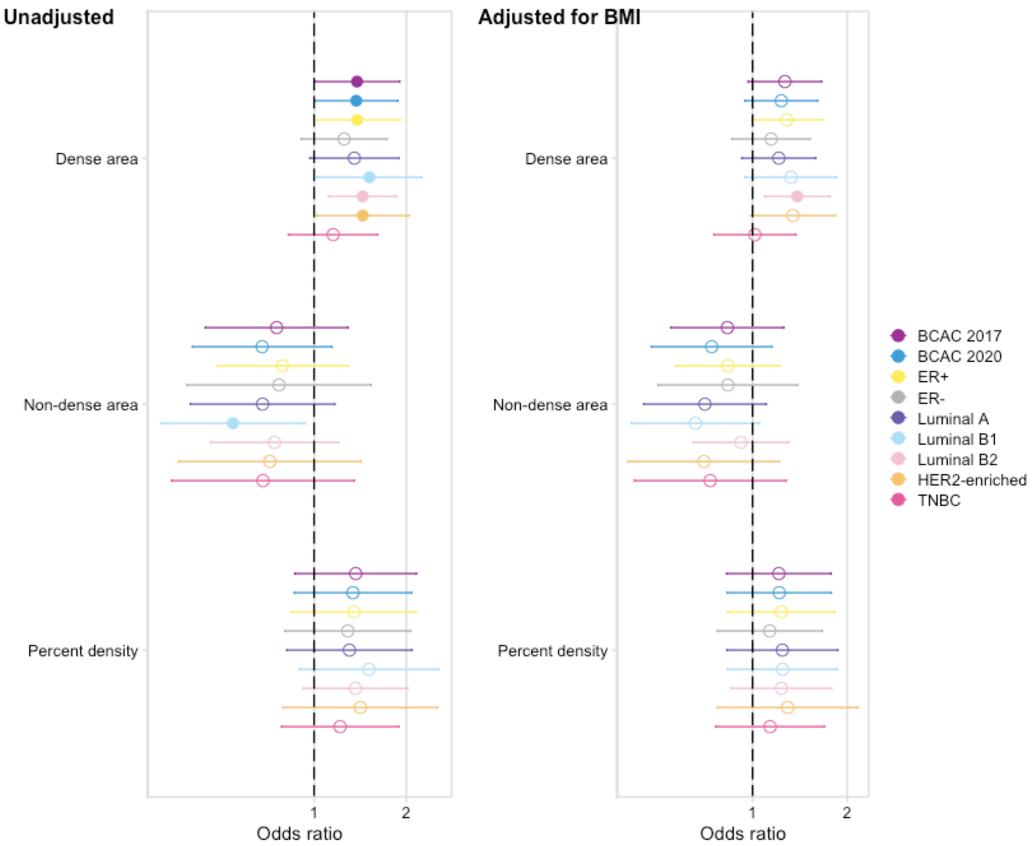

Supplementary Figure 3. The total (univariable MR) effect of MD phenotypes on breast cancer (overall and subtypes samples). **Unadjusted** plot (left) is the same as the results presented in Figure 3a in the paper, and is based on MD GWAS data unadjusted for BMI. **Adjusted for BMI** plot (right) is based on the data adjusted for BMI, as published in [1]. The instruments from both GWAS were extracted as described in Methods. The plots show the odds of breast cancer per SD higher MD phenotype. Bars indicate 95% confidence intervals around the point estimates from IVW analyses. The empty circle data points highlight the results where confidence intervals overlap the null.

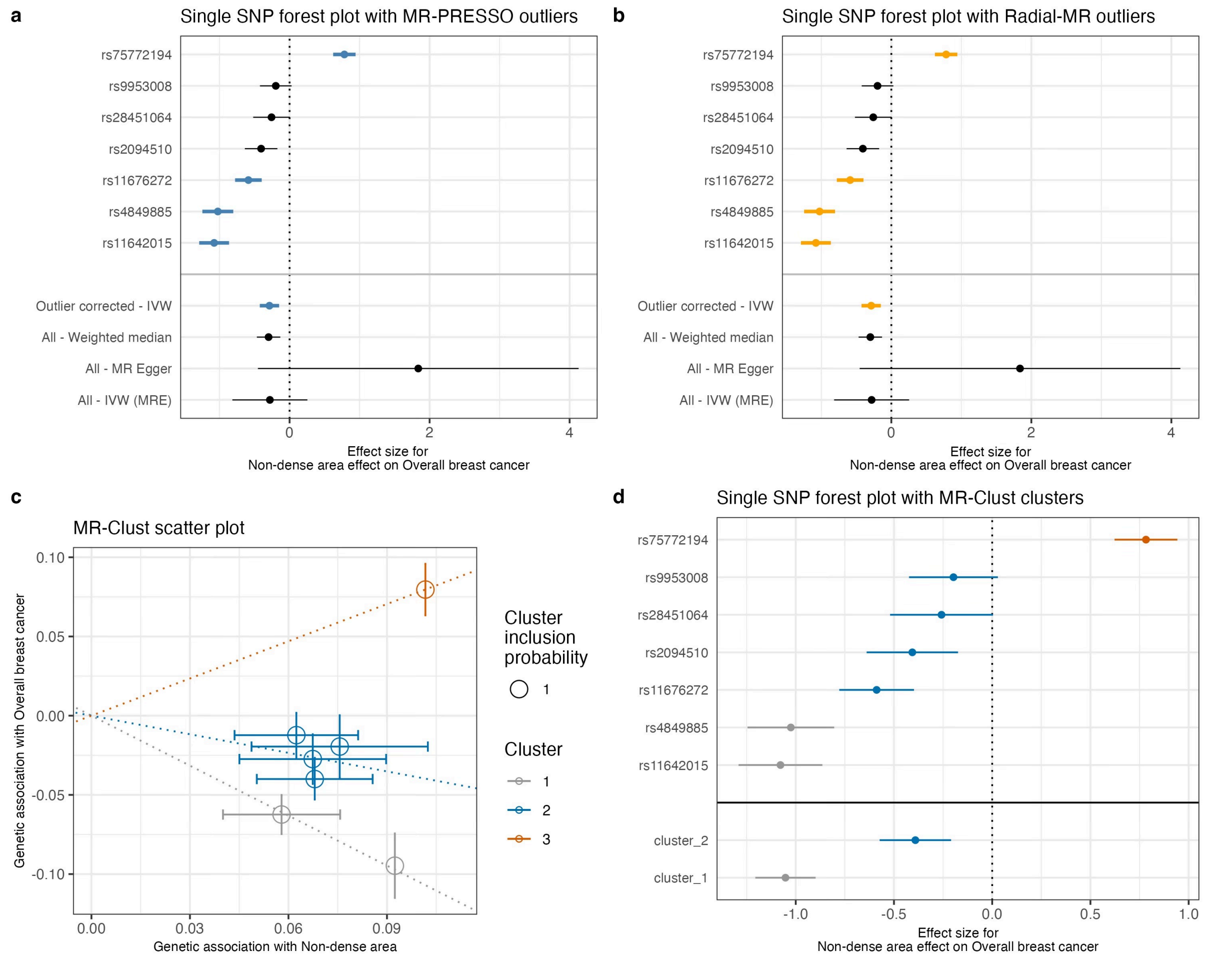

Supplementary Figure 4. Exploring the heterogeneity of genetic instruments of non-dense area phenotype on breast cancer (BCAC 2017 overall sample). (a) Single SNP forest plot (Wald Ratio estimates), with SNPs identified as outliers by MR-PRESSO marked in blue. The outlier corrected estimate is presented along with the standard MR methods estimates. **(b)** Single SNP forest plot with SNPs identified as outliers by Radial-MR marked in yellow. The outlier corrected estimate is presented along with the standard MR methods estimates. **(c)** MR-Clust scatter plot showing genetic association with dense area and breast cancer per SD change in dense area. Each genetic variant is represented by a point. Error bars are 95% confidence intervals of the Wald Ratio for each variant. Colours represent the clusters, and dotted lines represent the cluster means, the point size denotes cluster inclusion probability. The null cluster, coloured pink, relates to variants with null effect, whilst the black "junk cluster" are variants that were not assigned to any cluster. The error bars denote the standard error estimates of the Wald Ratio for each instrumental variable. **(d)** Single SNP forest plot with SNPs coloured by the cluster membership assigned by MR-Clust (using the same colours as in the scatter plot). The IVW MR estimates for each cluster are presented below single SNP estimates.

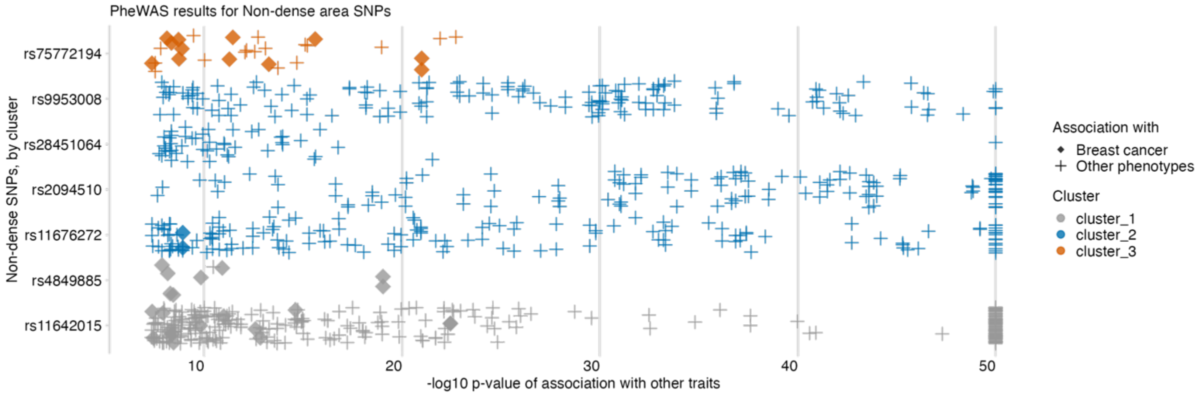

Supplementary Figure 5. PheWAS results for non-dense area phenotype genetic variants, ordered by SNP effect and cluster membership (from MR-Clust). The data points are other traits associated with a non-dense area SNPs (y-axis) at p-value <1e-08 (x-axis, -log10 scale, capped at value 50). The colour shows the cluster membership, in the same palette and order as in Figures 4c/4d. Data points represented by solid 'diamond' shapes are breast cancer outcomes; 'plus' shapes are all other traits.

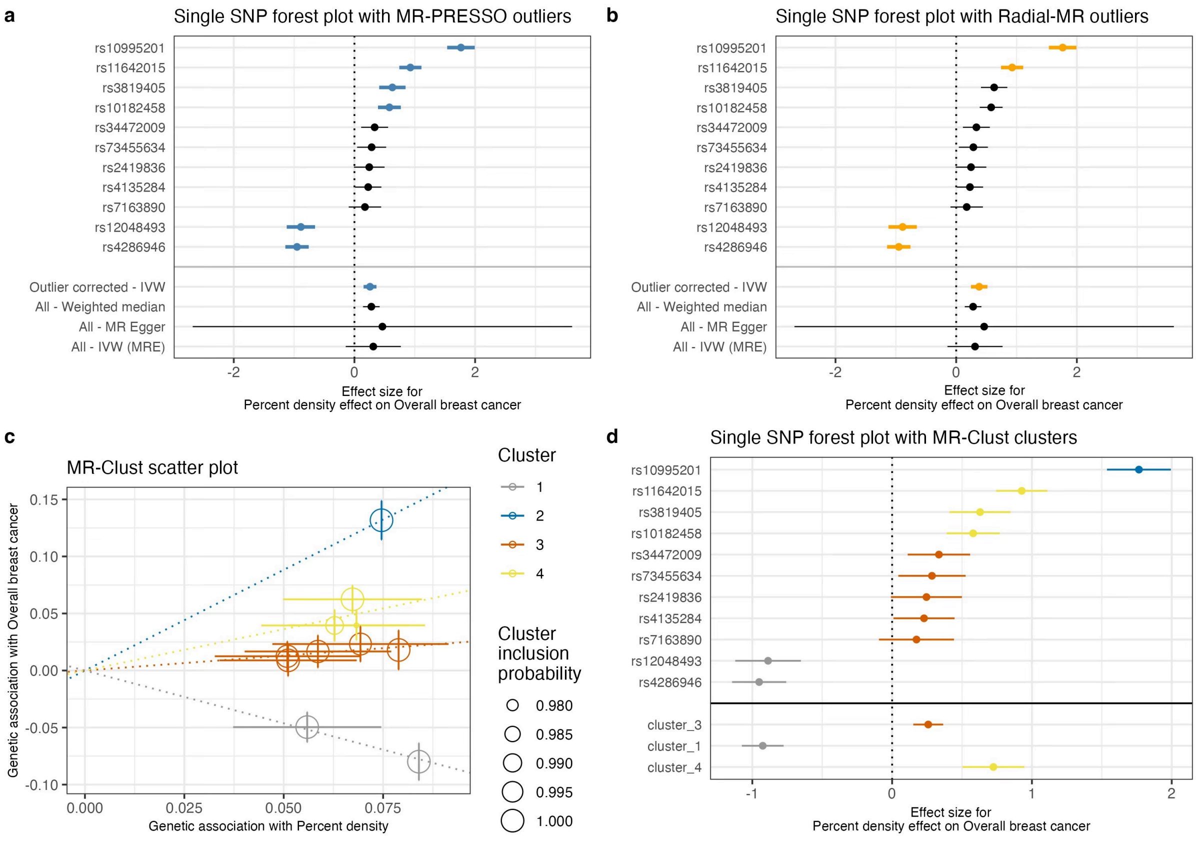

Supplementary Figure 6. Exploring the heterogeneity of genetic instruments of percent density phenotype on breast cancer (BCAC 2017 overall sample). (a) Single SNP forest plot (Wald Ratio estimates), with SNPs identified as outliers by MR-PRESSO marked in blue. The outlier corrected estimate is presented along with the standard MR methods estimates. **(b)** Single SNP forest plot with SNPs identified as outliers by Radial-MR marked in yellow. The outlier corrected estimate is presented along with the standard MR methods estimates. **(c)** MR-Clust scatter plot showing genetic association with dense area and breast cancer per SD change in dense area. Each genetic variant is represented by a point. Error bars are 95% confidence intervals of the Wald Ratio for each variant. Colours represent the clusters, and dotted lines represent the cluster means, the point size denotes cluster inclusion probability. The null cluster, coloured pink, relates to variants with null effect, whilst the black “junk cluster” are variants that were not assigned to any cluster. The error bars denote the standard error estimates of the Wald Ratio for each instrumental variable. **(d)** Single SNP forest plot with SNPs coloured by the cluster membership assigned by MR-Clust (using the same colours as in the scatter plot). The IVW MR estimates for each cluster are presented below single SNP estimates.

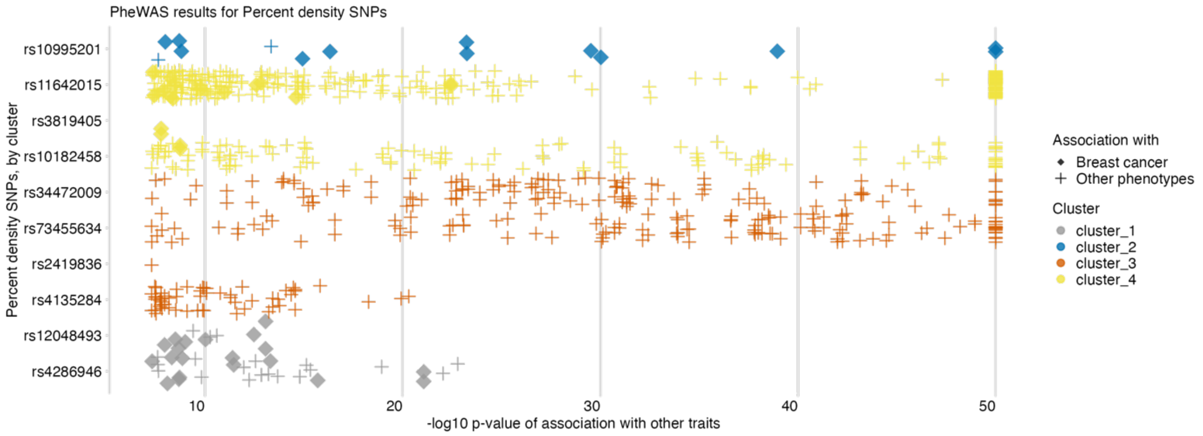

Supplementary Figure 7. PheWAS results for percent density phenotype genetic variants, ordered by SNP effect and cluster membership (from MR-Clust). The data points are other traits associated with a percent density SNPs (y-axis) at p-value <1e-08 (x-axis, -log10 scale, capped at value 50). The colour shows the cluster membership, in the same palette and order as in Figures 6c/6d. Data points represented by solid ‘diamond’ shapes are breast cancer outcomes; ‘plus’ shapes are all other traits.

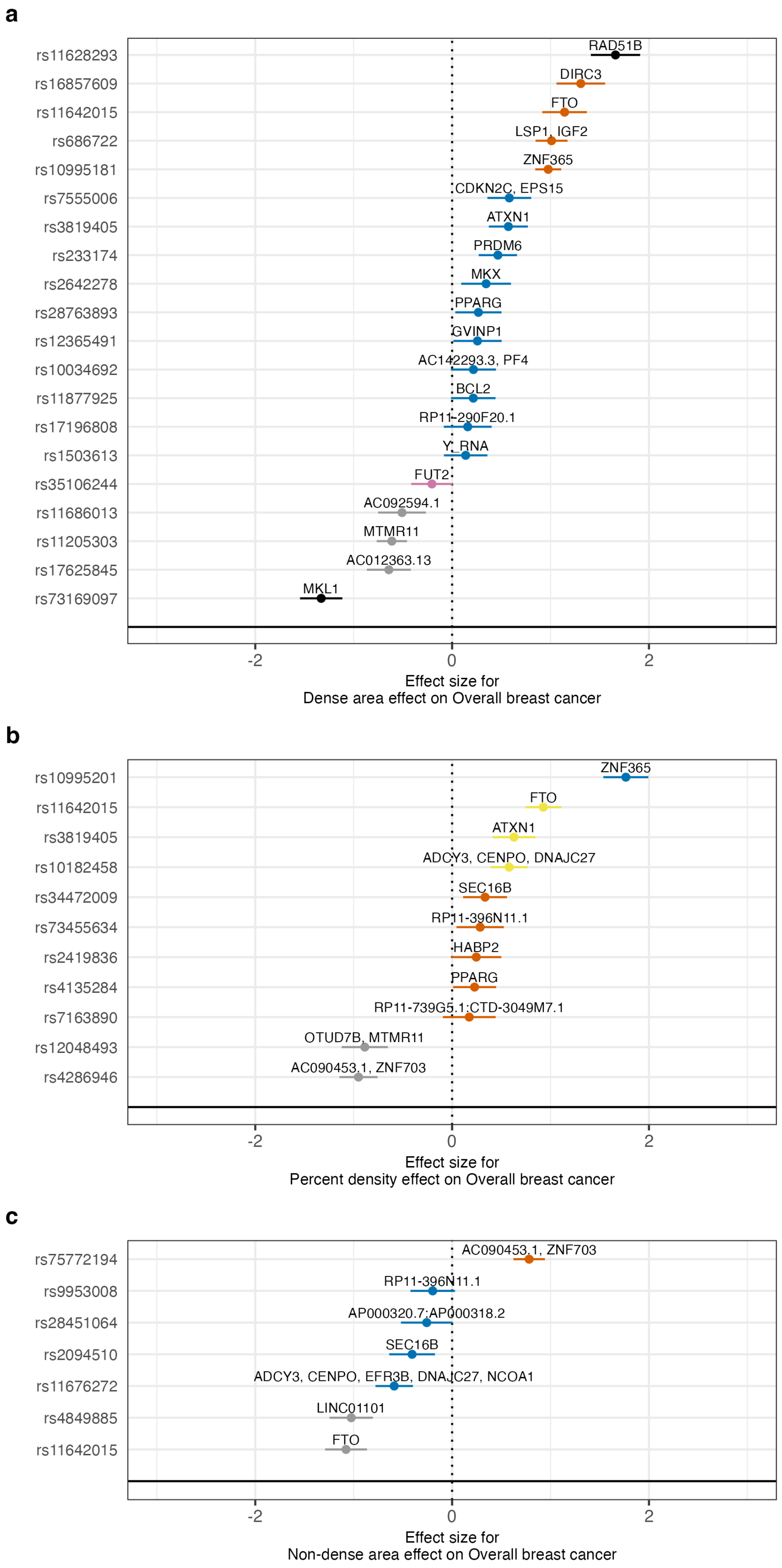

Supplementary Figure 8. (a) Dense area, (b) Percent density, (c) Non-dense area single SNP forest plot with SNPs coloured by the cluster membership assigned by MR-Clust, with each SNP mapped to a gene.

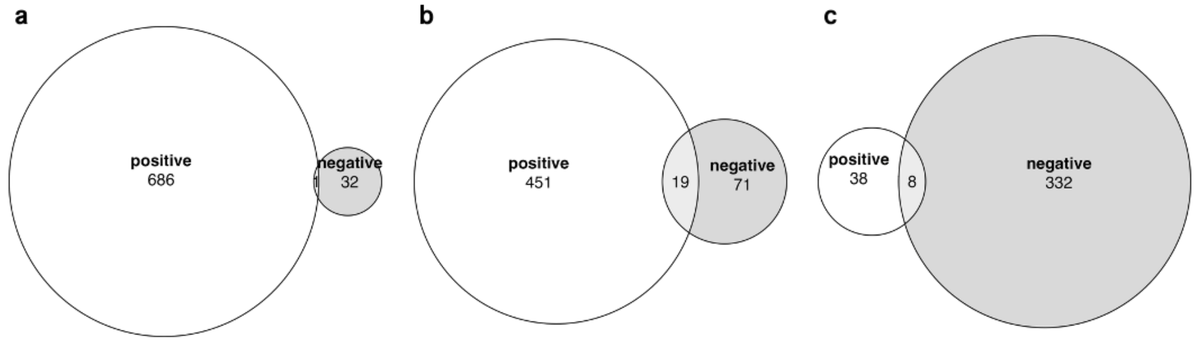

Supplementary Figure 9. (a) Dense area, (b) Percent density, (c) Non-dense area pathways counts Venn diagrams. For each phenotypes, instrument SNPs were mapped to genes and then pathways. SNPs/genes were splits into 'positive' and 'negative' effect direction sets, according to clustering results and individual SNPs estimates. The Venn diagrams show the number of unique pathways mapping to SNPs/genes in each effect direction set.

Supplementary Note 2. Mediation analysis calculation

Mediation analysis was carried out for childhood body size (exposure), dense area (mediator), and breast cancer BCAC 2017 overall sample (outcome).

We estimated the proportion of childhood body size effect on breast cancer that is mediated via dense area.

The total effect childhood body size effect on breast cancer:

b	se	pval	OR, 95%CIs
-0.411	0.0702	4.99E-09	0.66 [0.58: 0.76]

Analysis outline

See more details on mediation analysis in Supplementary Section E of our previous work (reference [7]).

1) Estimate 'indirect effect' of exposure on outcome via the mediator, using two methods:
   a. Difference method: **indirect effect = total effect – direct effect**
      ⇒ Estimate SE and CIs for the indirect effect using 'Propagation of Errors' method
      se.indirect = sqrt(se_total ^2 + se_direct^2)

   b. Product method: **indirect effect = exposure->mediator effect * mediator->outcome effect**
      ⇒ Estimate SE and CIs for the indirect effect using 'Delta' method
      se.indirect = sqrt(a_beta^2 * b_se^2 + b_beta^2 * a_se^2),
      where **a** is **exposure->mediator** and **b** is **mediator->outcome**

      mediator->outcome effect could be total effect or direct effect (from MVMR)

2) Estimate 'proportion mediated'
   ⇒ **proportion mediated = indirect effect / total effect *100%**

2.1 Calculation using all DA instruments

1a. Difference method

Indirect effect: –0.411 – (–0.195) = **–0.215**
SE = sqrt(0.0702^2 + 0.117 ^2) = 0.136
lo_ci = –0.215 – 1.96 * 0.136= –0.48
up_ci = –0.215 + 1.96 * 0.136= 0.05

–0.22 [–0.48: 0:05]

1b. Product method (using mediator->outcome direct effect)

Indirect effect: –0.62 * 0.37 = **–0.2294**
SE = sqrt(–0.62^2 * 0.069^2 + 0.37^2 * 0.068^2) = 0.0496
lo_ci = –0.2294 –1.96 * 0.0496 = –0.326
up_ci = –0.2294 + 1.96 * 0.0496= –0.132

–0.23 [–0.33: –0.13]

2. Proportion mediated (% mediated)

Product method

% mediated = **–0.2294** / –0.411 * 100% = **55.8%**
% mediated (upper CI) = –0.326 / –0.411 * 100% = 79.3%
% mediated (lower CI) = –0.132 / –0.411 * 100% = 32.1%

56% [32% - 79%]

Difference method

% mediated = **–0.215** / –0.411 * 100% = **52.3%**

CIs for % mediated are not calculated here as the effect CIs overlap the null.

2.2 Calculation using <u>DA instruments from cluster_2 only</u> (i.e. outlier-corrected IVW)

First, MVMR of childhood body size and dense area (cluster_2 instruments only, n=10) was performed to estimate the direct effects:

Childhood body size: beta -0.223, se 0.0826, p-value 6.91e-03
Dense area: beta 0.301, se 0.0613, p-value 9.30e-07

<u>1a. Difference method</u>

Indirect effect: –0.411 – (–0.223) = **–0.188**

SE = sqrt(0.0702^2 + 0.0826^2) = 0.108

lo_ci = –0.188 – 1.96 * 0.108= -0.399
up_ci = –0.188 + 1.96 * 0.108= 0.024

–0.22 [–0.48: 0:05] (based on all instruments, from Note 2.1)
–0.19 [–0.40: 0:02] (using outlier-corrected estimates)

<u>1b. Product method</u> (using mediator->outcome direct effect)

Indirect effect: –0.62 * 0.30 = **–0.186**

SE = sqrt(–0.62^2 * 0.0613^2 + 0.30^2 * 0.068^2) = 0.0431

lo_ci = –0.186 –1.96 * 0.0431 = –0.270
up_ci = –0.186 + 1.96 * 0.0431= –0.102

–0.23 [–0.33: –0.13] (based on all instruments, from Note 2.1)
–0.19 [–0.27: –0.10] (using outlier-corrected estimates)

<u>2. Proportion mediated (% mediated)</u>

Product method

% mediated = **–0.186** / –0.411 * 100% = **45.3%**
% mediated (upper CI) = –0.27 / –0.411 * 100% = 66%
% mediated (lower CI) = –0.10 / –0.411 * 100% = 24%
45% [24% - 66%]

Difference method

% mediated = **–0.187** / –0.411 * 100% = **45.7%**

CIs for % mediated are not calculated here as the effect CIs overlap the null.

Supplementary Note 3. Outlier-corrected IVW for breast cancer subtypes

Figure 10 (next page). **Exploring the heterogeneity of genetic instruments of dense phenotype on breast cancer subtypes.** Single SNP forest plots with SNPs identified as outliers by Radial-MR marked in yellow. The outlier corrected estimate is presented along with the standard MR methods estimates. Error bars are 95% confidence intervals of the Wald Ratio for each variant. The x-axis of each plot is labelled with the breast cancer subtype. The SNPs are ordered according to their order in the main results (overall sample in panel 'a', which is the same as Figure 4b).

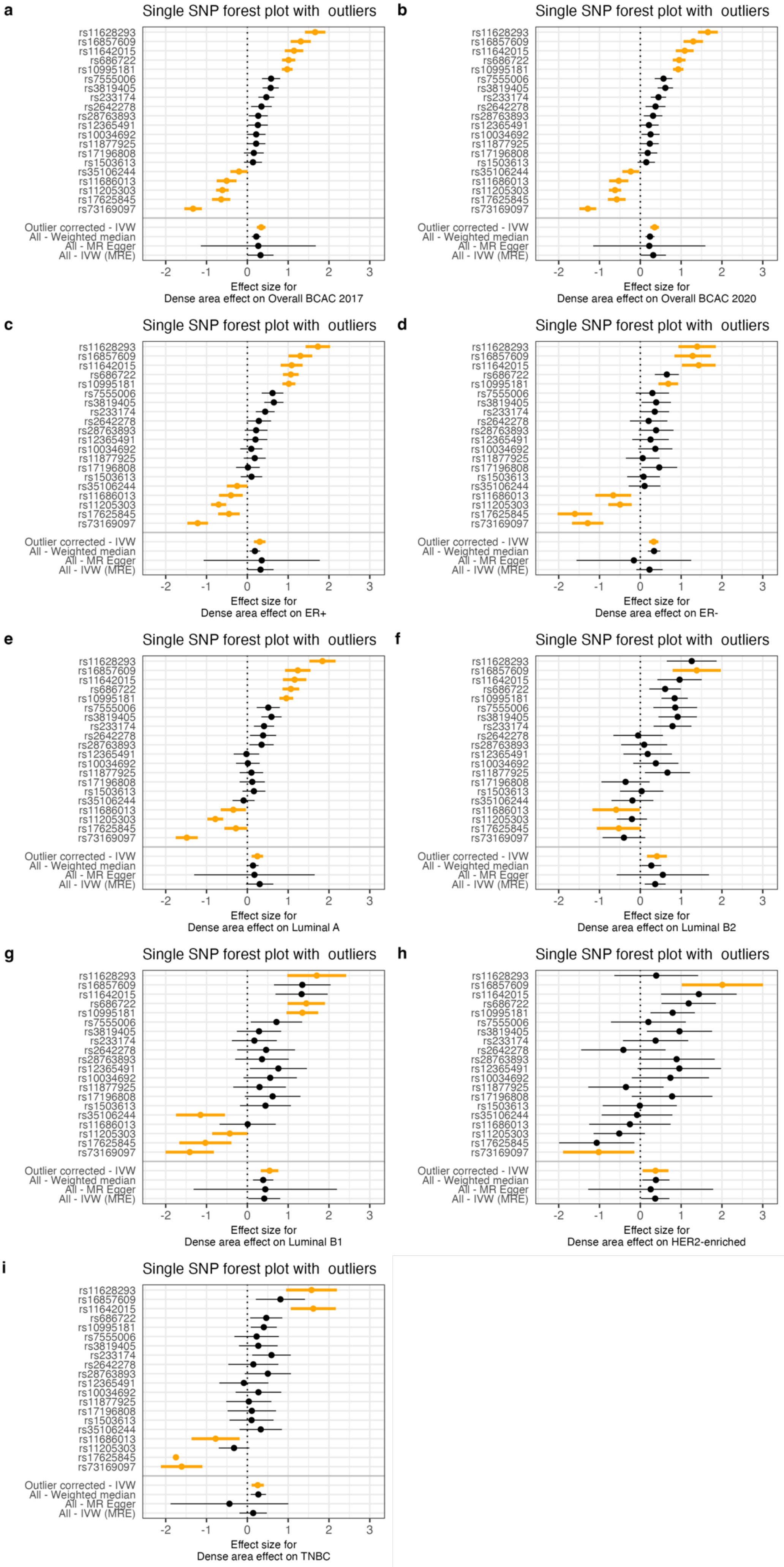
a
Single SNP forest plot with outliers
rs11628293
rs16857609
rs11642015
rs686722
rs10995181
rs7555006
rs3819405
rs233174
rs2642278
rs28763893
rs12365491
rs10034692
rs11877925
rs17196808
rs1503613
rs35106244
rs11686013
rs11205303
rs17625845
rs73169097
Outlier corrected - IVW
All - Weighted median
All - MR Egger
All - IVW (MRE)
-2 -1 0 1 2 3
Effect size for
Dense area effect on Overall BCAC 2017
b
Single SNP forest plot with outliers
Effect size for
Dense area effect on Overall BCAC 2020
c
Single SNP forest plot with outliers
Effect size for
Dense area effect on ER+
d
Single SNP forest plot with outliers
Effect size for
Dense area effect on ER-
e
Single SNP forest plot with outliers
Effect size for
Dense area effect on Luminal A
f
Single SNP forest plot with outliers
Effect size for
Dense area effect on Luminal B2
g
Single SNP forest plot with outliers
Effect size for
Dense area effect on Luminal B1
h
Single SNP forest plot with outliers
Effect size for
Dense area effect on HER2-enriched
i
Single SNP forest plot with outliers
Effect size for
Dense area effect on TNBC

Supplementary references

[1] W. Sieh *et al.*, "Identification of 31 loci for mammographic density phenotypes and their associations with breast cancer risk," *Nat. Commun.*, vol. 11, no. 1, Dec. 2020.

[2] K. Michailidou *et al.*, "Association analysis identifies 65 new breast cancer risk loci," 2017.

[3] H. Zhang *et al.*, "Genome-wide association study identifies 32 novel breast cancer susceptibility loci from overall and subtype-specific analyses," *Nat. Genet.*

[4] E. Bouras *et al.*, "Circulating inflammatory cytokines and risk of five cancers: a Mendelian randomization analysis," *BMC Med.*, vol. 20, no. 1, pp. 1–15, 2022.

[5] F. P. Hartwig, K. Tilling, G. Davey Smith, D. A. Lawlor, and M. C. Borges, "Bias in two-sample Mendelian randomization when using heritable covariable-adjusted summary associations," *Int. J. Epidemiol.*, vol. 50, no. 5, pp. 1639–1650, 2021.

[6] J. Gilbody, M. C. Borges, G. Davey Smith, and E. Sanderson, "Multivariable MR can mitigate bias in two-sample MR using covariable-adjusted summary associations," *medRxiv*, p. 2022.07.19.22277803, Jul. 2022.

[7] M. Vabistsevits, G. Davey Smith, E. Sanderson, T. G. Richardson, B. Lloyd-Lewis, and R. C. Richmond, "Deciphering how early life adiposity influences breast cancer risk using Mendelian randomization," *Commun. Biol.*, vol. 5, no. 1, 2022.
