# Supplementary material for: Mammographic density mediates the protective effect of early-life body size on breast cancer risk
Source: Nat Commun. 2024 May 13;15:4021. doi: 10.1038/s41467-024-48105-7 (PMC11091136; doi:10.1038/s41467-024-48105-7)
Supplement: Supplementary file 3 — Description of Additional Supplementary Files [file 41467_2024_48105_MOESM3_ESM.pdf]

Description of Additional Supplementary Files:

Supplementary Data 1: Univariable MR (total effect, IVW) of childhood and adult body size on MD phenotypes

Supplementary Data 2: Sensitivity analysis: Univariable MR (total effect, IVW) of childhood and adult body size on MD phenotypes

Supplementary Data 3: MVMR (direct effect) of childhood and adult body size together on MD phenotypes

Supplementary Data 4: Sensitivity analysis: MVMR (direct effect) of childhood and adult body size together on MD phenotypes

Supplementary Data 5: Univariable MR (total effect, IVW) of age at menarche on MD phenotypes

Supplementary Data 6: Sensitivity analysis: Univariable MR (total effect, IVW) of age at menarche on MD phenotypes

Supplementary Data 7: MVMR (direct effect) of age at menarche and childhood body size on MD phenotypes

Supplementary Data 8: Sensitivity analysis: MVMR (direct effect) of age at menarche and childhood body size on MD phenotypes

Supplementary Data 9: Univariable MR (total effect, IVW) of MD phenotypes on breast cancer

Supplementary Data 10: Sensitivity analysis: Univariable MR (total effect, IVW) of MD phenotypes on breast cancer

Supplementary Data 11: MVMR (direct effect) of MD phenotypes and childhood body size on breast cancer

Supplementary Data 12: Sensitivity analysis: MVMR (direct effect) of MD phenotypes and childhood body size on breast cancer

Supplementary Data 13: Univariable MR (total effect, IVW) of childhood body size on breast cancer

Supplementary Data 14: MR PRESSO results

Supplementary Data 15: MR-Radial results

Supplementary Data 16: MR-Clust results

Supplementary Data 17: DA PheWAS

Supplementary Data 18: NDA PheWAS

Supplementary Data 19: PD PheWAS

Supplementary Data 20: SNP to gene mapping: DA

Supplementary Data 21: SNP to gene mapping: NDA

Supplementary Data 22: SNP to gene mapping: PD

Supplementary Data 23: Genes to pathways mapping: DA

Supplementary Data 24: Genes to pathways mapping: PDA

Supplementary Data 25: Genes to pathways mapping: PD

Supplementary Data 26: Univariable MR (total effect, IVW) of childhood and adult body size on MD phenotypes (adj for BMI)

Supplementary Data 27: Sensitivity analysis: Univariable MR (total effect, IVW) of childhood and adult body size on MD phenotypes (adj for BMI)

Supplementary Data 28: MVMR (direct effect) of childhood and adult body size together on MD phenotypes (adj for BMI)

Supplementary Data 29: Sensitivity analysis: MVMR (direct effect) of childhood and adult body size together on MD phenotypes (adj for BMI)

Supplementary Data 30: Univariable MR (total effect, IVW) of age at menarche on MD phenotypes (adj for BMI)

Supplementary Data 31: Sensitivity analysis: Univariable MR (total effect, IVW) of age at menarche on MD phenotypes (adj for BMI)

Supplementary Data 32: MVMR (direct effect) of age at menarche and childhood body size on MD phenotypes (adj for BMI)

Supplementary Data 33: Sensitivity analysis: MVMR (direct effect) of age at menarche and childhood body size on MD phenotypes (adj for BMI)

Supplementary Data 34: Univariable MR (total effect, IVW) of MD phenotypes (adj for BMI) on breast cancer

Supplementary Data 35: Sensitivity analysis: Univariable MR (total effect, IVW) of MD phenotypes (adj for BMI) on breast cancer

Supplementary Data 36: MVMR (direct effect) of MD phenotypes (adj for BMI) and childhood body size on breast cancer

Supplementary Data 37: Sensitivity analysis: MVMR (direct effect) of MD phenotypes (adj for BMI) and childhood body size on breast cancer
