# Supplementary material for: Mammographic density mediates the protective effect of early-life body size on breast cancer risk
Source: Nat Commun. 2024 May 13;15:4021. doi: 10.1038/s41467-024-48105-7 (PMC11091136; doi:10.1038/s41467-024-48105-7)
Supplement: Supplementary file 5 — Reporting Summary [file 41467_2024_48105_MOESM5_ESM.pdf]

nature portfolio

Corresponding author(s): Marina Vabistsevits

Last updated by author(s): Feb 28, 2024

Reporting Summary

Nature Portfolio wishes to improve the reproducibility of the work that we publish. This form provides structure for consistency and transparency in reporting. For further information on Nature Portfolio policies, see our Editorial Policies and the Editorial Policy Checklist.

Statistics

For all statistical analyses, confirm that the following items are present in the figure legend, table legend, main text, or Methods section.

n/a	Confirmed	
[ ]	[x]	The exact sample size (*n*) for each experimental group/condition, given as a discrete number and unit of measurement
[x]	[ ]	A statement on whether measurements were taken from distinct samples or whether the same sample was measured repeatedly
[ ]	[x]	The statistical test(s) used AND whether they are one- or two-sided<br>*Only common tests should be described solely by name; describe more complex techniques in the Methods section.*
[ ]	[x]	A description of all covariates tested
[ ]	[x]	A description of any assumptions or corrections, such as tests of normality and adjustment for multiple comparisons
[ ]	[x]	A full description of the statistical parameters including central tendency (e.g. means) or other basic estimates (e.g. regression coefficient) AND variation (e.g. standard deviation) or associated estimates of uncertainty (e.g. confidence intervals)
[ ]	[x]	For null hypothesis testing, the test statistic (e.g. *F*, *t*, *r*) with confidence intervals, effect sizes, degrees of freedom and *P* value noted<br>*Give P values as exact values whenever suitable.*
[x]	[ ]	For Bayesian analysis, information on the choice of priors and Markov chain Monte Carlo settings
[x]	[ ]	For hierarchical and complex designs, identification of the appropriate level for tests and full reporting of outcomes
[x]	[ ]	Estimates of effect sizes (e.g. Cohen's *d*, Pearson's *r*), indicating how they were calculated

Our web collection on statistics for biologists contains articles on many of the points above.

Software and code

Policy information about availability of computer code

Data collection	No software was used for data collection
Data analysis	All analyses were conducted using R (v4.2.1). Univariable MR analyses and sensitivity tests were performed using the TwoSampleMR R package (v0.5.6), which was also used for accessing GWAS summary data deposited in OpenGWAS (gwas.mrcieu.ac.uk). Multivariable MR was carried out using the MVMR R package (version 0.2).<br>All code is available at: https://github.com/mvab/mammographic_density_mr

For manuscripts utilizing custom algorithms or software that are central to the research but not yet described in published literature, software must be made available to editors and reviewers. We strongly encourage code deposition in a community repository (e.g. GitHub). See the Nature Portfolio guidelines for submitting code & software for further information.

Data

Policy information about availability of data

All manuscripts must include a data availability statement. This statement should provide the following information, where applicable:

- Accession codes, unique identifiers, or web links for publicly available datasets
- A description of any restrictions on data availability
- For clinical datasets or third party data, please ensure that the statement adheres to our policy

The GWAS data for BCAC 2017 breast cancer (IDs: ieu-a-1126, ieu-a-1127, ieu-a-1128), and childhood body size (ID: ieu-b-510), and age at menarche (ID: ukb-

b-3768) is accessible from the OpenGWAS database (https://gwas.mrcieu.ac.uk). The BCAC 2020 molecular subtype data is available at https://bcac.ccge.medschl.cam.ac.uk/bcacdata/ oncoarray/oncoarray-and-combined-summary-result/. Childhood and adult body size GWAS data was published in ref [5] (Richardson et al 2020).

This study uses data from a GWAS of mammographic density (ref [32], Sieh et al 2020). The RPGEH genotype data are available upon application to the KP Research Bank (https://researchbank.kaiserpermanente.org/). Additional relevant information is available from the authors upon reasonable request.

Research involving human participants, their data, or biological material

Policy information about studies with human participants or human data. See also policy information about sex, gender (identity/presentation), and sexual orientation and race, ethnicity and racism.

Reporting on sex and gender	This study was performed using summary data based on female sex data only (sex as a biological attribute). The study used genetic data, and sample sex verification is commonly performed as a part of genotyping QC. Hence, the presented findings apply only to biological females.
Reporting on race, ethnicity, or other socially relevant groupings	All used GWAS datsesets were produced using European ancestry cohorts, as this is the only data available to investigate our research question with sufficient power.
Population characteristics	Body size GWAS were performed in UK Biobank, which is an ongoing large-scale prospective cohort study with genetic and phenotypic data collected on approximately 500,000 individuals from across the UK, aged 40-69. Further details are available in Bycroft et al 2018 “The UK Biobank resource with deep phenotyping and genomic data”<br><br>Mammographic density GWAS was performed in non-Hispanic white women aged between 40-74 years from a larger population-based study, RPGEH (Research Program on Genes, Environment and Health), administered by Kaiser Permanente Northern California (KPNC) Division of Research. Further details in "Characterizing race/ethnicity and genetic ancestry for 100,000 subjects in the genetic epidemiology research on adult health and aging (GERA) cohort" by Banda et al 2015
Recruitment	UK Biobank participants were recruited between the years 2006 and 2010 from across the UK, aged 40-69 at recruitment. A rich variety of phenotypic and health-related information is available on each participant, including biological measurements, lifestyle indicators, biomarkers in blood and urine, and imaging of the body and brain. Further details are available in Bycroft et al 2018 “The UK Biobank resource with deep phenotyping and genomic data”
Ethics oversight	UK Biobank has received ethical approval from the UK National Health Service’s National Research Ethics Service (ref 11/NW/0382). At recruitment, all participants gave informed consent to participate in the UK Biobank and be followed-up with. The analysis of other datasets was done on summary statistics and does not require additional ethical approval.

Note that full information on the approval of the study protocol must also be provided in the manuscript.

Field-specific reporting

Please select the one below that is the best fit for your research. If you are not sure, read the appropriate sections before making your selection.

[x] Life sciences [ ] Behavioural & social sciences [ ] Ecological, evolutionary & environmental sciences

For a reference copy of the document with all sections, see nature.com/documents/nr-reporting-summary-flat.pdf

Life sciences study design

All studies must disclose on these points even when the disclosure is negative.

Sample size	Our study analyses summary level data from published GWAS, and therefore sample sizes were predetermined as described in their corresponding publications. All sample sizes are reported in Methods/Data Sources, along with their corresponding original publications and summaries of study designs. No sample size calculations were conducted as a part of this analysis. The selected GWAS datasets have sufficient samples sizes to achieve good instruments strength (measured by F-statistics).
Data exclusions	As summary data was used, no individuals exclusions were performed.
Replication	NA - the only data that could be used for replication currently does not provide effect sizes publicly (MD BCAC by Chen et al 2022)
Randomization	Our approach (Mendelian randomization) is based on the quasi randomization of genetic variants at conception - randomization occurred naturally as those genetic variants were used to instrument the exposure.
Blinding	Genetic association testing and Mendelian randomization does not require blinding.

Reporting for specific materials, systems and methods

We require information from authors about some types of materials, experimental systems and methods used in many studies. Here, indicate whether each material, system or method listed is relevant to your study. If you are not sure if a list item applies to your research, read the appropriate section before selecting a response.

Materials & experimental systems

n/a	Involved in the study
☒	☐ Antibodies
☒	☐ Eukaryotic cell lines
☒	☐ Palaeontology and archaeology
☒	☐ Animals and other organisms
☒	☐ Clinical data
☒	☐ Dual use research of concern
☒	☐ Plants

Methods

n/a	Involved in the study
☒	☐ ChIP-seq
☒	☐ Flow cytometry
☒	☐ MRI-based neuroimaging
